# Development and Temporal Validation of Machine Learning Models for Hyponatremia Risk in Community-Dwelling Older Adults: A Nationwide Claims-Based Study

**DOI:** 10.3390/jcm15135072

**Published:** 2026-06-29

**Authors:** Hee-Jae Lee, Kwanghee Jun

**Affiliations:** 1 College of Pharmacy, Gyeongsang National University, Jinju-si 52828, Republic of Korea; heejaelee@gnu.ac.kr; 2 Research Institute of Pharmaceutical Sciences, Gyeongsang National University, Jinju-si 52828, Republic of Korea

**Keywords:** aged, clinical decision support systems, hyponatremia, machine learning, predictive modeling, polypharmacy, risk prediction

## Abstract

**Background/Objectives**: Hyponatremia is a clinically important electrolyte disorder in older adults, yet early identification is hindered by complex, non-linear interactions between comorbidities and polypharmacy. This study aimed to develop and externally validate a machine learning (ML) prediction model for hyponatremia risk using nationwide claims data, focusing on medication patterns and clinical features. **Methods**: A retrospective cohort study was conducted using the South Korean Health Insurance Review and Assessment Service-Aged Patient Sample (HIRA-APS). Data from 2017 to 2018 were used for development, and 2019 data for temporal external validation (age ≥ 65). SHapley Additive exPlanations (SHAP)-based recursive feature elimination identified 33 high-impact predictors from 60 clinical features. Six ML algorithms, including LightGBM and CatBoost, were trained with 1:4 case–control matching and evaluated for discrimination and calibration. **Results**: The development and validation cohorts included 4810 and 648,586 patients, respectively. All models showed comparable discriminative performance (area under the receiver operating characteristic curve [AUROC] 0.741–0.746), with LightGBM achieving the highest (AUROC 0.746; 95% confidence interval (CI) 0.728–0.763). The models had very high negative predictive values (>0.999) for ruling out low-risk individuals. Tree-based ensemble matched linear models in discrimination but achieved better calibration. **Conclusions**: These validated, interpretable ML models can serve as clinical decision support tools that rule out low-risk patients and prioritize monitoring for high-risk individuals. Across sociodemographic subgroups, calibration was maintained after recalibration, whereas discrimination was lower in the oldest, most comorbid, frailest, highest-medication-burden, and lowest-socioeconomic groups—a gap to address before equitable deployment.

## 1. Introduction

Proactively identifying and managing the underlying risk factors for major geriatric syndromes, such as frailty and falls, remains a central challenge in the primary care of older adults [1,2,3]. Among the many contributors to this vulnerability, hyponatremia, a common yet often underrecognized electrolyte disorder, is gaining attention for its substantial clinical impact and its prevalence, which rises steeply with the intensity of care among older adults. A systematic review pooling four decades of data reported a prevalence of mild hyponatremia of approximately 22% on geriatric wards and 19% in nursing homes, far exceeding that in general hospital wards (~6%), establishing it as a major burden across older-adult care settings [4,5,6]. A narrative review reported that hyponatremia increases the risk of falls by approximately 2.14-fold and fractures by 2.00-fold [7,8]. This clinical significance extends to even mild, often overlooked cases (130–135 mmol/L), which have been associated with a 3.02-fold increase in fall risk [9,10,11], a 1.37-fold increase in mortality [12,13,14], and measurable cognitive-screening decrements [15]. Thus, hyponatremia has been quantitatively established not as a benign laboratory finding, but as an associated risk factor that consistently increases the clinical burden across various patient populations. Nevertheless, identifying at-risk individuals in community and primary care settings presents a significant challenge. Vague, nonspecific symptoms are often dismissed, and the high prevalence of multimorbidity and polypharmacy leaves a critical blind spot in the management of vulnerable older adults [16,17].

In hospitalized patients, routine laboratory monitoring facilitates the timely identification of hyponatremia, and reported prevalence is higher among inpatients, rising with the intensity of care [5]. In community and primary care settings, detection is more difficult because many cases are asymptomatic or present with nonspecific complaints, and consultation times are often limited. Mild chronic hyponatremia—commonly described as 130–134 mmol/L persisting for >48 h—may lack overt neurological signs, instead manifesting subtle psychomotor deficits such as impaired gait and attention [18,19]. In older adults with multimorbidity, these features are easily attributed to aging or comorbid conditions, and iatrogenic contributors such as thiazide diuretics and selective serotonin reuptake inhibitors (SSRIs) further complicate evaluation [20,21,22,23]. Because general checkups do not typically include routine electrolyte screening, and repeated testing may be impractical in short consultations, some cases are likely to remain unrecognized until laboratory assessment is performed. Given these detection challenges, a targeted approach that combines a high index of clinical suspicion with rational, evidence-based testing strategies is warranted for older adults.

Current screening instruments, including the American Geriatrics Society (AGS) Beers Criteria^®^ and the Screening Tool of Older Persons’ Prescriptions/Screening Tool to Alert to Right Treatment (STOPP/START), are instrumental in identifying potentially inappropriate medications; however, these frameworks do not primarily encompass individualized quantitative risk assessment and are intended to serve as adjuncts to, rather than substitutes for, shared clinical decision-making [24,25]. This gap is illustrated by randomized trials such as SENATOR and OPERAM. SENATOR found poor uptake (approximately 15%) and failed to reduce adverse drug reactions [26,27], while OPERAM showed no significant reduction in drug-related hospital admissions despite successfully addressing inappropriate prescribing [28,29]. While emerging machine learning models can capture non-linear interactions, their clinical utility remains to be established via multi-site external validation and transportability assessments across heterogeneous care settings [30,31,32]. Most existing models have been developed in single, hospital-based cohorts with limited external validation, reinforcing concerns about out-of-distribution performance in community-dwelling populations. In addition, the literature has predominantly prioritized treatment-response prediction (e.g., sodium correction trajectories and overcorrection risk) rather than proactive risk stratification for primary prevention in the community. Although nationwide administrative claims data enable longitudinal medication exposure analysis, predictive modeling remains sparse. This is further complicated by the inherent constraints of claims-based outcome ascertainment, in which hyponatremia captured through diagnostic codes may be incompletely recorded relative to laboratory-confirmed events; we address this through an explicit operational outcome definition and revisit it as a source of potential misclassification in the Discussion [33,34]. This gap between alerting and clinical impact is particularly consequential for community-dwelling older adults, where infrequent electrolyte monitoring intersects with iatrogenic risks from common agents like thiazides and SSRIs, compounding detection challenges [35]. These detection gaps are unlikely to fall evenly across the older population: risk concentrates in the oldest-old and in those with the heaviest comorbidity and polypharmacy burden (the same subgroups whose limited access to frequent laboratory monitoring compounds the chance that hyponatremia goes unrecognized), so an equitable tool must perform consistently across these strata, an aspect this study explicitly revisits in evaluating model fairness [36,37]. Therefore, there is an urgent need for externally validated, generalizable prediction tools for this evidence-to-practice gap.

This study aimed to develop and externally validate a machine learning (ML)-based prediction model for hyponatremia in community-dwelling older adults using nationwide claims data. Specifically, we sought to: (1) incorporate features capturing medication use patterns and polypharmacy; (2) utilize ML approaches with SHAP-based interpretability to capture complex interactions while maintaining clinical transparency; and (3) perform temporal external validation across consecutive years to assess generalizability. The resulting model is intended to serve as a clinical decision support tool for primary-care clinicians (prescribers and pharmacists performing medication review) for proactive risk identification [38,39]. Using claims-based administrative information, it supports targeted monitoring and safer prescribing in older adults.

## 2. Materials and Methods

### 2.1. Data Source and Population

We conducted a retrospective cohort study using data from the Health Insurance Review and Assessment Service-Aged Patient Sample (HIRA-APS) from 2017 and 2018, while the 2019 dataset was separately constructed to serve as a temporally independent cohort for external validation. The HIRA-APS is a nationally representative claims database comprising approximately 10% of the older adult population in South Korea, derived via stratified random sampling. Because Korea’s public health coverage is mandatory and effectively universal—comprising National Health Insurance (over 97% of the population), the Medical Aid program, and the Veterans’ medical benefit scheme, all adjudicated by HIRA [40,41]—diagnoses and prescriptions are recorded as a billing requirement, distinguishing this source from voluntary registries [42,43,44]. It contains comprehensive, de-identified information on diagnoses, medication prescriptions, and healthcare utilization.

The study cohort included patients aged 65 years or older, with the index date defined as the date of their first outpatient medical visit between June 1 and September 30 of each year. From the eligible population, patients were excluded based on the following criteria: (1) no medication prescriptions recorded during the 5 months before the cohort entry date; or (2) a diagnosis of hyponatremia during the same 5-month period before cohort entry.

This study received ethical approval from the Institutional Review Board (IRB) of Gyeongsang National University on 4 July 2025 (IRB No. GIRB-G25-NX-0068). The requirement for informed consent was waived, as the dataset is fully de-identified.

### 2.2. Outcome Definition and Follow-Up

The primary outcome was the first incident of hyponatremia during follow-up. As laboratory serum sodium values are not captured in the HIRA-APS claims database, direct biochemical ascertainment was not feasible; we therefore established a code- and prescription-based operational definition. An incident case of hyponatremia was defined as the first occurrence of either (1) a prescription for tolvaptan or 3% hypertonic saline or (2) a primary or secondary diagnosis of hyponatremia (International Classification of Diseases, 10th Revision [ICD-10] code E87.1) or syndrome of inappropriate antidiuretic hormone secretion (SIADH) (E22.2). The definition, therefore, captures clinically recognized hyponatremia—cases that enter the care pathway through a recorded diagnosis or directed treatment, the actionable target for a primary-care decision-support tool; mild, untreated cases ascertainable only by laboratory screening are not captured, as we note in the Limitations. Because this under-ascertainment misclassifies cases as non-cases, it dilutes the case–control contrast and biases discrimination conservatively rather than inflating it.

This study was not prospectively registered. As a retrospective analysis of pre-existing de-identified administrative claims, it was not eligible for clinical-trial registration; the analytic plan, including cohort construction, outcome definition, feature set, and validation strategy, was pre-specified internally before model development; the full pre-specified analytic plan is available from the corresponding author on reasonable request.

**Figure 1:**
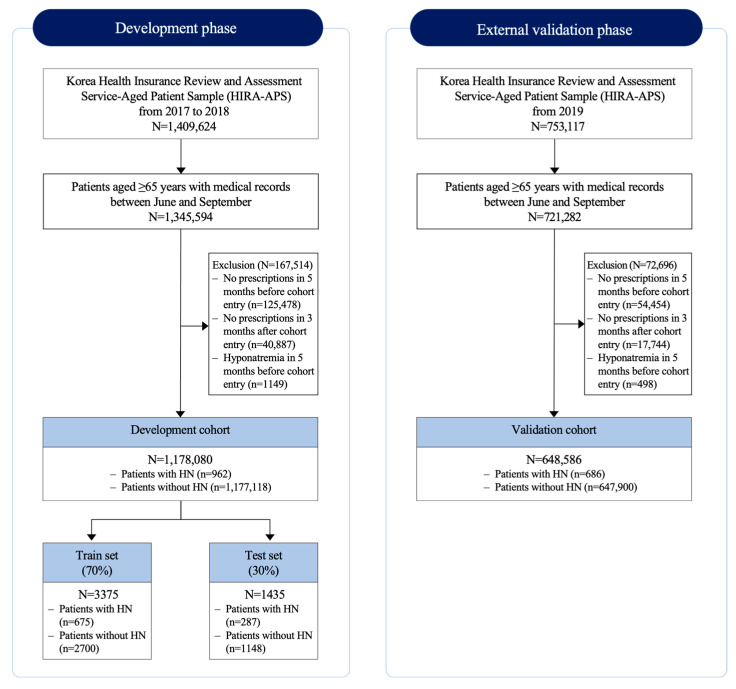
Flowchart for patient selection.

Patients were followed from their index date until the first occurrence of a study outcome, death, or the end of the study period (December 31 of each year). To ensure continuous healthcare utilization, patients without any prescription record during the initial 3 months of follow-up were excluded from the final analysis. A flowchart of the cohort selection process is provided in Figure 1.

To construct a predictive model aligned with clinical decision-making, we defined patient characteristics across two distinct, purposive timeframes relative to the index date. First, features representing the patient’s current health status were ascertained as of the index date. This point-in-time assessment included demographics, comorbidities, polypharmacy status, and scores for the Charlson Comorbidity Index (CCI) and frailty. Second, to capture recent clinical medication exposure, we established a 3-month look-back period preceding the index date. This period was used to define medication-related features, including concurrent use of different hyponatremia-inducing medication (HIM) classes and the usage pattern for individual HIMs (i.e., new user, persistent user, or non-user).

### 2.3. Candidate Features

Based on literature reviews, the AGS Beers Criteria 2019 and 2023, and recent meta-analyses, we identified and extracted 60 candidate features potentially associated with hyponatremia. These features were ascertained using the specific timeframes relative to the index date as previously described, capturing the patient’s current health status and recent medication exposure.

Medication exposure was characterized by identifying 11 classes of HIMs (Supplementary Table S1). Three distinct usage patterns were defined for these HIMs: new use (first prescription during the look-back period), persistent use (≥2 consecutive prescriptions), and concurrent use (≥1 day of overlap between two different HIMs). To better reflect real-world adherence, prescription duration was extended by 25% (days supplied × 1.25), corresponding to an assumed adherence of approximately 80% (1/0.80 = 1.25), consistent with adherence levels reported for chronic cardiovascular medication in Korean nationwide claims studies [45,46]. A full list of candidate features is provided in Supplementary Table S2.

### 2.4. Model Development and Validation

#### 2.4.1. Data Splitting

Model development was based on the 2017–2018 cohort. To address the significant class imbalance inherent in the raw data, we first constructed a matched cohort through 1:4 case–control matching on age and sex. This 1:4 ratio followed established pharmacoepidemiologic practice, balancing statistical power against computational efficiency. The matched dataset was then partitioned by stratified random sampling into a training set (70%) and an internal hold-out test set (30%). Matching was applied only during development to manage class imbalance; the 2019 temporal validation used the full, unmatched cohort at the true population prevalence (≈0.11%), so the reported validation performance reflects the real-world case mix.

#### 2.4.2. Data Preprocessing

Numerical features were standardized with a standard scaler, and categorical features were encoded using one-hot encoding to improve model stability and performance. No candidate feature contained missing values, since all variables were derived from systematically recorded claims data. This preprocessing pipeline was applied uniformly across all patients, without any subgroup-specific transformation.

#### 2.4.3. Feature Selection

To identify the most informative and stable feature set, we applied SHAP (SHapley Additive Explanations)-based RFECV (Recursive Feature Elimination with Cross-Validation), which yielded a final set of 33 features for model training. Sample size adequacy was then confirmed against the events-per-variable (EPV) criterion: with 962 hyponatremia cases for these 33 features, the development cohort yielded an EPV of 29.15, well above the recommended minimum of 10 for reliable machine learning model development.

#### 2.4.4. Model Training and Performance Evaluation

During 10-fold cross-validation on the training set, the Synthetic Minority Over-sampling Technique (SMOTE) was applied only to the training folds; this prevented data leakage and further mitigated residual imbalance within each fold while leaving the validation folds free of synthetic data. Six machine learning algorithms were trained: Logistic Regression (LR), LASSO, Random Forest, LightGBM, XGBoost, and CatBoost. Hyperparameters for each model were optimized using 10-fold cross-validation on the training set, with the objective of maximizing the area under the receiver operating characteristic curve (AUROC). We employed GridSearch for linear models and Bayesian optimization with Optuna for tree-based models.

The performance of the trained models was evaluated on the unseen internal test set. We assessed discrimination using the AUROC and the area under the precision-recall curve (AUPRC), and calibration using the Brier score and calibration plots. To evaluate potential clinical utility, an optimal probability threshold was determined using Youden’s index, at which point we calculated accuracy, sensitivity, specificity, positive predictive value (PPV), negative predictive value (NPV), and the F1-score. The 95% confidence intervals (CI) for the AUROC were derived from 1000 bootstrap resamples. Threshold-based operating characteristics were used to characterize clinical utility.

#### 2.4.5. External Validation

To assess the model’s generalizability and temporal robustness, an external validation was performed on the 2019 cohort. This approach tested the model’s performance in a real-world setting, simulating prospective deployment. The models were applied without recalibration to assess transportability and were evaluated using the same input features and optimized hyperparameter settings. Because development used a 1:4-matched cohort with enriched prevalence, we additionally recalibrated the validation predictions to the cohort prevalence by a logit intercept correction and quantified calibration with the calibration slope and calibration-in-the-large; this monotone transformation does not affect discrimination.

#### 2.4.6. Decision-Curve Analysis

To evaluate clinical utility beyond discrimination and calibration, we performed decision-curve analysis on the recalibrated LightGBM predictions in the 2019 temporal-validation cohort. Net benefit was calculated as NB = (TP/N) − (FP/N) × [*p_t_*/(1 − *p_t_*)], where TP and FP are the numbers of true- and false-positive classifications at threshold probability *p_t_* and N is the cohort size and was compared against the default strategies of treating all and treating no patients [47]. Because the outcome prevalence was low (≈0.11%), net benefit was examined over the correspondingly low threshold-probability range relevant to a rule-out application (approximately 0.1–0.5%). The analysis was repeated on the uncalibrated predictions to isolate the effect of recalibration. Threshold-specific 95% CIs for net benefit were obtained from 1000 bootstrap resamples.

#### 2.4.7. Subgroup and Fairness Analysis

To assess the robustness and equity of model performance, we evaluated the LightGBM model in the 2019 temporal-validation cohort across the following clinical strata: sex, age group, insurance type, Charlson Comorbidity Index category, frailty category, and medication burden. Insurance type (health insurance, Medical Aid, and National Meritorious Service) served as a validated proxy for socioeconomic status, as direct socioeconomic indicators are not recorded in HIRA-APS; the Medical Aid category identifies the low-income population. Within each stratum, discrimination was summarized as the AUROC with DeLong’s 95% CI and calibration as the post-recalibration calibration-in-the-large (CITL) on the log-odds scale. Estimates from strata with fewer than 30 outcome events were considered imprecise and were not interpreted. Formal group-fairness metrics (e.g., equalized odds and subgroup-stratified decision-curve analysis) were beyond the scope of this study and are identified as necessary pre-deployment work.

#### 2.4.8. Statistical Analysis and Software

For comparison of cohort characteristics, we used Chi-square tests for categorical variables to calculate the *p*-value and standardized mean difference. Data management and preprocessing were performed using SAS Enterprise Guide 8.3 (SAS Institute Inc., Cary, NC, USA). Model development and validation were conducted in a Python 3.8 environment. Key libraries included scikit-learn (v0.24.2), pandas (v1.3.0), numpy (v1.20.3), matplotlib (v3.4.2), LightGBM (v3.2.1), XGBoost (v1.4.2), and CatBoost (v0.26.1).

## 3. Results

### 3.1. Baseline Characteristics of the Study Population

The development cohort comprised 4810 patients (962 with hyponatremia and 3848 matched controls). The external validation cohort included 648,586 patients. Detailed baseline characteristics for both cohorts are presented in Table 1.

Patients with hyponatremia showed greater clinical vulnerability than controls across both cohorts. The hyponatremia group was significantly older, with 50.9% of patients aged ≥75 years compared to 46.5% of controls in the development cohort (standardized mean difference [SMD] = 0.105). This pattern was consistent in the validation cohort (71.1% vs. 42.9%; SMD = 0.652). The burden of severe comorbidity (CCI score ≥ 5) was more than twice as high in cases versus controls in both the development (15.5% vs. 6.7%; SMD = 0.456) and validation cohorts (13.7% vs. 5.2%; SMD = 0.423). Similarly, a high degree of frailty (frailty score > 5) was more prevalent in the hyponatremia group (Development: 31.8% vs. 13.7%, SMD = 0.540; Validation: 23.2% vs. 8.8%, SMD = 0.499).

Medication-related risk factors were pronounced and showed a clear relationship. The prevalence of polypharmacy (≥10 medications) was more than double in cases compared to controls in both the development (55.6% vs. 27.0%; SMD = 0.682) and validation cohorts (52.5% vs. 27.4%; SMD = 0.615). Regarding HIMs, the persistent use of agents like thiazide diuretics (25.6% vs. 15.2%; SMD = 0.261) and the concurrent use of certain HIM pairs were more frequent in cases (e.g., thiazide–SIADH-inducing drugs: 11.8% vs. 3.6% in the validation cohort). Conversely, the proportion of patients unexposed to any HIMs was substantially lower in the hyponatremia group (28.6% vs. 48.9% in the validation cohort).

### 3.2. Model Performance

**Figure 2:**
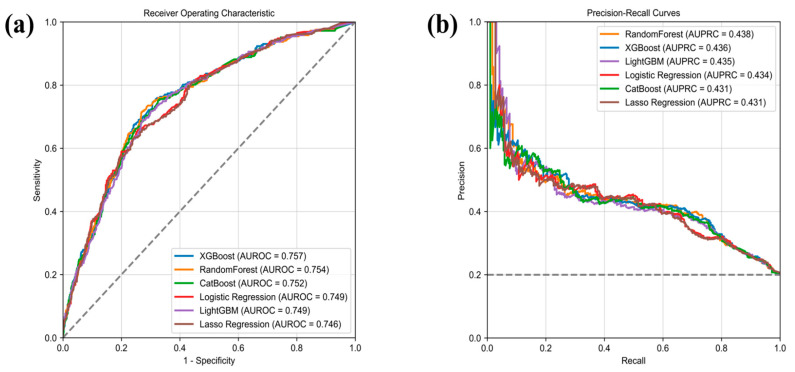
Predictive performance of each model. (**a**) Receiver operating characteristic (ROC) curves and (**b**) precision–recall (PR) curves. AUPRC, area under precision–recall curve; AUROC, area under receiver operating characteristic. The dashed diagonal line in (**a**) denotes the reference line of a non-discriminative classifier (AUROC = 0.5); the dashed horizontal line in (**b**) indicates the baseline precision corresponding to the outcome prevalence.

In the internal validation, all six models showed comparable discriminative performance. The AUROC values ranged from 0.746 to 0.757 (Table 2). The high degree of overlap in the ROC curves visually confirms this minimal difference in performance. This finding was further supported by the Precision-Recall Curve (PRC) analysis, where all models yielded similar AUPRC values, ranging from 0.431 to 0.438 (Figure 2).

The models’ discrimination was preserved in the temporal external validation, with AUROCs ranging from 0.741 to 0.746. The LightGBM model achieved the highest performance in this cohort (AUROC 0.746; 95% CI 0.728–0.763). Due to the low prevalence of hyponatremia in this real-world setting (approximately 0.11%), the PPV was low for all models (<0.01). Conversely, the NPV was very high (>0.999), suggesting the models’ primary utility as screening tools for ruling out low-risk patients.

### 3.3. Model Calibration

**Figure 3:**
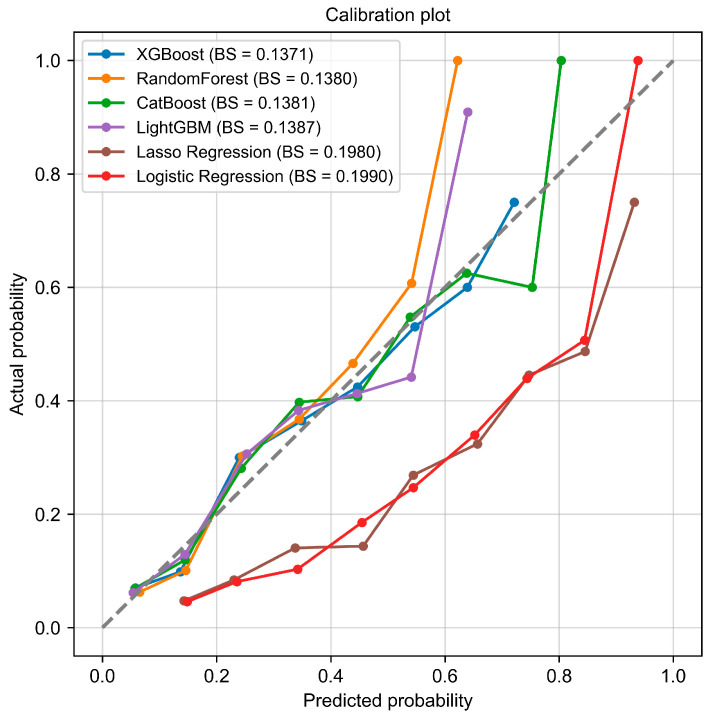
Calibration plots of the six predictive models. BS, Brier score.

Calibration analysis revealed that tree-based models provided more reliable risk estimates than linear models. In internal testing, tree-based models demonstrated superior calibration with substantially lower Brier scores (0.137–0.139) compared to linear models (0.198–0.199). This was visually confirmed by the calibration plot, where the curves for tree-based models more closely followed the ideal diagonal line, indicating better agreement between predicted probabilities and observed outcomes (Figure 3). This performance gap persisted in external validation, where tree-based models (Brier scores 0.040–0.043) again outperformed linear models (0.189–0.191).

On the full 2019 validation cohort, the LightGBM calibration slope was 1.01 (95% CI, 0.92–1.11), indicating correctly scaled predictions (no over- or under-dispersion), whereas the calibration-in-the-large was −5.36 (95% CI, −5.44 to −5.29) on the log-odds scale—reflecting the expected base-rate shift from 1:4 matched development (mean predicted risk 0.16 vs. observed prevalence 0.0011). A single logit-intercept recalibration to the local prevalence removed this shift (calibration-in-the-large ≈ 0) while leaving discrimination unchanged by construction (AUROC 0.746); cross-validated Platt scaling, used as a monotonicity check, gave an essentially identical AUROC (0.744) and a calibration slope of 0.99 (Supplementary Figure S2).

### 3.4. Model Interpretation

**Figure 4:**
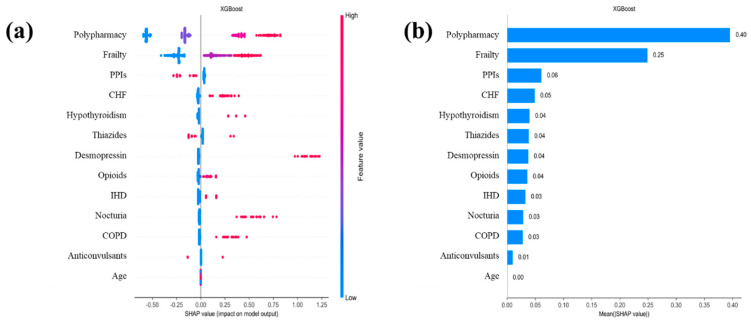
Interpretability of the XGBoost model based on SHAP values. (**a**) SHAP summary plot and (**b**) feature importance based on mean absolute SHAP values. SHAP, SHapley Additive exPlanations.

To understand the clinical drivers of the predictions, the XGBoost model was interpreted using SHAP analysis as a representative example from the high-performing tree-based models; XGBoost was selected for the primary interpretation because it attained the highest discrimination on the development test set, and the tree-based models converged on the same dominant predictors, so the choice of interpretation model is not consequential for the conclusions. The SHAP summary plot identified polypharmacy (number of medications) and frailty score as the most influential predictors, followed by other comorbidity and medication features (Figure 4). The plot confirmed that higher values for these features consistently drove the model’s prediction toward a higher risk of hyponatremia, indicating that the model relied on clinically plausible risk factors. Interpretability analyses for the LightGBM and CatBoost models yielded similar feature importance patterns (Supplementary Figure S1).

### 3.5. Decision-Curve Analysis

**Figure 5:**
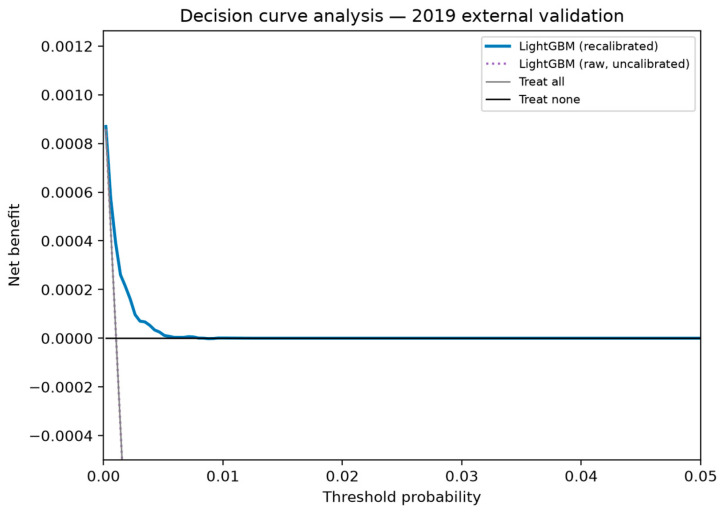
Decision-curve analysis on the 2019 temporal-validation cohort (recalibrated LightGBM). Net benefit versus threshold probability, with treat-all and treat-none reference strategies.

Decision-curve analysis on the 2019 cohort showed that, after recalibration, the model’s net benefit exceeded both default strategies (treat-all and treat-none) across the narrow low threshold-probability range relevant at this prevalence (approximately 0.1–0.2%); for example, at a 0.2% threshold, the net benefit was 2.0 × 10^−4^ (95% CI, 1.4–2.5 × 10^−4^; ≈2 net true-positive detections per 10,000 screened) versus net harm for treat-all, whereas at thresholds of approximately 0.5% and above, it was indistinguishable from treating no one. The uncalibrated model showed no net benefit at any threshold, consistent with a low-threshold rule-out use rather than high-risk confirmation (Figure 5).

### 3.6. Subgroup Performance

To assess robustness and equity, we evaluated the LightGBM model on the 2019 temporal-validation cohort across clinically defined strata—sex, age, insurance type (a validated proxy for socioeconomic status), Charlson comorbidity index (CCI), frailty, and medication burden—reporting discrimination (AUROC with DeLong 95% CI) and post-recalibration calibration-in-the-large (CITL); the full results are presented in Supplementary Table S3. After recalibration, calibration was maintained across all strata, with only small residual CITL (largest ≈ −0.43 on the log-odds scale in the lowest-prevalence age 65–69 stratum); because a single global intercept cannot match every stratum’s base rate, these residuals would be removed by stratum-specific recalibration. Discrimination, by contrast, was not uniform: it was comparable between the sexes but lowest across the highest-risk strata (age ≥ 75, 0.675; CCI ≥ 5, 0.665; Medical Aid, 0.669; highest medication burden, 0.696), relative to the overall cohort (0.746). Because this decrement was shared across all of the highest-risk strata rather than confined to the socioeconomic group, it is most consistent with reduced within-stratum case-mix heterogeneity (range restriction)—in which age and comorbidity, themselves the dominant risk drivers, leave a smaller residual discriminative signal—rather than a socioeconomic-specific effect. For the low-socioeconomic (Medical Aid) group, the 95% CI (0.612–0.722) lay just below, without overlapping, that of the health-insurance majority (0.747; 95% CI, 0.727–0.766); given this narrow margin, we interpret the difference as hypothesis-generating evidence of an equity concern requiring formal fairness evaluation rather than a confirmed disparity.

## 4. Discussion

In this nationwide study of community-dwelling older adults, we developed and temporally validated machine learning models for predicting hyponatremia. The models demonstrated clinically useful discrimination (AUROC ≈ 0.75), which was preserved on temporal external validation. Within each cohort, tree-based algorithms achieved lower Brier scores than the linear models, indicating better probabilistic calibration at a fixed outcome prevalence. SHAP analysis confirmed that the model’s principal predictors—polypharmacy, frailty, and comorbidity burden—were clinically plausible. Given the low outcome prevalence, the models yielded a low PPV but a consistently high NPV (>0.999), indicating their primary clinical utility as a screening tool to effectively rule out low-risk individuals.

While most prior prediction models for hyponatremia have focused on hospitalized patients, our study addresses the distinct context of primary care for community-dwelling older adults [48,49]. It moves beyond the simple flagging of potentially inappropriate medications, as seen in tools like the Beers Criteria or in large-scale trials such as OPERAM and SENATOR, which showed limited impact on clinical outcomes. By integrating complex medication patterns to provide an individualized, quantitative risk score, our model offers a potential pathway to bridge the well-established ‘alert-to-impact’ gap in clinical decision support [50].

Prescribing-safety frameworks such as the Beers Criteria and STOPP/START represent medication risk as drug-level binary appropriateness flags, yet large deprescribing trials (OPERAM, SENATOR) show that alert-only interventions rarely improve hard endpoints, a gap attributable not only to alert fatigue and limited clinician uptake but also to the representational coarseness of binary flags [51]. Using SHAP-based recursive feature elimination over patient burden, comorbidities, and 11 HIM classes, our model selected a parsimonious 33-feature subset. Among the selected features, thiazides, desmopressin, and anticonvulsants independently converge on the antidiuretic hormone (ADH) axis [52], a clinically coherent profile we interpret as a predictive association rather than a causal mechanism. Clinically, this supports a shift from drug-by-drug review to profile-level prioritization, enabling targeted electrolyte monitoring of high-risk medication profiles in primary care.

The model’s principal clinical value lies in optimizing screening strategies through its high NPV. A practical application would be its integration into electronic health records (EHRs) to support a resource-efficient workflow: extending electrolyte monitoring intervals for low-risk patients while targeting high-risk individuals for intensive medication review. Importantly, the model’s output is a risk probability, not a diagnosis. The statistically derived Youden’s index threshold may not align with all clinical goals; therefore, implementing a purpose-driven thresholding strategy, particularly for ruling out, would be essential for its role as a clinical decision support tool. Operationally, the model is clinician-facing and consumes only automatically extracted administrative fields, requiring no manual data entry or specialist input to generate a risk score. Because development inputs were complete by construction (claims data with no missingness), the model’s behavior under degraded or incomplete inputs at the point of care has not been characterized and would require evaluation before deployment.

Our study has several strengths. A primary advantage is the model’s consistent performance within a highly heterogeneous, nationwide administrative database. While recent studies, such as that by van Orten-Luiten et al. (2025), have reported similar discrimination in specific primary care cohorts, achieving this in a more challenging, real-world national dataset demonstrates the model’s stability and generalizability [53]. This was a deliberate design choice; unlike previous models developed in single centers or for niche subpopulations, our model was intentionally built for universal applicability to community-dwelling older adults.

Methodologically, our approach was data-driven and reproducible. Instead of arbitrarily selecting predictors, we employed a data-driven feature selection process from a large pool of candidates, ensuring objectivity. The consistent performance across multiple machine learning algorithms further suggests that the predictive signal is genuine and not an artifact of a single method. We also moved beyond simple dichotomous drug exposure variables by creating granular features that capture nuanced medication use patterns (e.g., new, persistent, or concurrent use), which reflects a more detailed pharmaco-epidemiological approach.

Our model is oriented toward earlier risk identification. It flags under-recognized at-risk individuals for proactive monitoring, complementing rather than replacing the prognostic models used in secondary or tertiary care. Consistent with its operational outcome, the model’s evidenced value is rule-out: the high negative predictive value supports de-intensifying monitoring in low-risk patients, whereas the low positive predictive value and narrow net-benefit range do not support its use to confirm high-risk cases. Finally, because the model’s inputs are limited to routinely collected administrative claims that are uniformly recorded across Korea’s mandatory, near-universal public coverage, rather than to resource-intensive electronic health record infrastructure available only at well-resourced centers, it is, in principle, deployable at the national scale and could help narrow disparities in access to preventive screening [54,55]. We frame this as a design-level potential rather than a demonstrated outcome, since equitable real-world performance would require the formal subgroup and fairness evaluation outlined below.

We acknowledge several limitations. First, our operational outcome definition, reliant on administrative codes and proxy prescriptions, is subject to potential misclassification bias and likely underestimates the incidence of mild, untreated hyponatremia that never enters the clinical pathway; the model therefore predicts clinically recognized rather than biochemically defined hyponatremia [56]. Second, claims data do not capture several granular clinical variables, including volemic status, prescribing indication, dietary and fluid factors, and over-the-counter medication use, any of which could act as residual confounders. The dietary and fluid factors include habitual sodium and fluid intake, as well as the sodium restriction commonly advised in chronic kidney disease and congestive heart failure. Medication exposure, although adjusted to an assumed ~80% adherence, reflects dispensing rather than confirmed ingestion. Third, the model was developed in a single-country, ethnically homogeneous population, and its generalizability to other healthcare systems and ethnicities requires further validation. Fourth, the development and validation cohorts were constructed asymmetrically: the model was trained on a 1:4 case–control matched cohort with an artificially enriched outcome prevalence (~20%), whereas temporal external validation used the unmatched real-world cohort with a true prevalence of ~0.11%. This asymmetry is responsible for the very low absolute Brier scores and PPV observed in validation and means that the Youden-derived operating point is not directly transportable; deployment would require threshold re-derivation at the target-setting prevalence and prospective recalibration [57,58]. Finally, a formal fairness and subgroup analysis, not originally pre-specified, has now been performed (Supplementary Table S3): after recalibration, calibration was maintained across age, sex, socioeconomic, comorbidity, frailty, and medication-burden strata, whereas discrimination was lower in the oldest (AUROC 0.68 for ≥ 75 y), most comorbid (0.67 for CCI ≥ 5), frailest (0.69 for frailty > 5), highest-medication-burden (0.70), and lowest-socioeconomic (0.67 for Medical Aid) subgroups; this lower discrimination is shared across the highest-risk strata rather than specific to socioeconomic status, and closing this discrimination gap—together with formal group-fairness metrics—is a necessary step before equitable deployment.

## 5. Conclusions

Using only routinely collected administrative claims, a machine learning model can reliably identify community-dwelling older adults at low risk of hyponatremia, supporting a resource-efficient strategy that relaxes monitoring for low-risk patients while prioritizing review of high-risk individuals. Its evidenced value is therefore a rule-out rather than a confirmation of high-risk cases. Clinical translation will require prospective validation against laboratory-confirmed outcomes in electronic health records, alongside assessment of transportability and recalibration across diverse international settings.

## Figures and Tables

**Table 1:** Baseline characteristics of the study participants in the development and validation cohorts.

Variables, *N* (%)	Development Cohort					Validation Cohort				
	Total Patients	HN Patients	Non-HN Patients	*p*-Value	SMD	Total Patients	HN Patients	Non-HN Patients	*p*-Value	SMD
	(*N* = 4810)	(*N* = 962)	(*N* = 3848)			(*N* = 648,586)	*(N* = 686)	(*N* = 647,900)		
**Age, years**				0.015	0.105				<0.001	0.652
65–69	1386 (28.8)	243 (25.3)	1143 (29.7)			205,307 (31.7)	69 (10.1)	205,238 (31.7)		
70–74	1144 (23.8)	229 (23.8)	915 (23.8)			165,082 (25.5)	129 (18.8)	164,953 (25.5)		
≥75	2280 (47.4)	490 (50.9)	1790 (46.5)			278,197 (42.9)	488 (71.1)	277,709 (42.9)		
**Sex, Female**	2922 (60.7)	601 (62.5)	2321 (60.3)	0.235	0.044	376,859 (58.1)	447 (65.2)	376,412 (58.1)	<0.001	0.146
**Insurance type**				0.085	0.056				<0.001	0.149
Health insurance	4311 (89.6)	844 (87.7)	3467 (90.1)			593,445 (91.5)	589 (85.9)	592,856 (91.5)		
Medical aid	424 (8.8)	102 (10.6)	322 (8.4)			46,001 (7.1)	82 (12.0)	45,919 (7.1)		
National meritorious service	75 (1.6)	16 (1.7)	59 (1.5)			9140 (1.4)	15 (2.2)	9125 (1.4)		
**CCI score**				<0.001	0.456				<0.001	0.423
0–2	3332 (69.3)	499 (51.9)	2833 (73.6)			503,497 (77.6)	405 (59.0)	503,092 (77.6)		
3–4	1073 (22.3)	314 (32.6)	759 (19.7)			111,536 (17.2)	187 (27.3)	111,349 (17.2)		
≥5	405 (8.4)	149 (15.5)	256 (6.7)			33,553 (5.2)	94 (13.7)	33,459 (5.2)		
**Frailty score**				<0.001	0.540				<0.001	0.499
0 ≤ score ≤ 2	2780 (57.8)	370 (38.5)	2410 (62.6)			432,972 (66.8)	308 (44.9)	432,664 (66.8)		
2 < score ≤ 5	1196 (24.9)	286 (29.7)	910 (23.6)			158,350 (24.4)	219 (31.9)	158,131 (24.4)		
>5	834 (17.3)	306 (31.8)	528 (13.7)			57,264 (8.8)	159 (23.2)	57,105 (8.8)		
**Comorbidities**										
Alcoholism	55 (1.1)	17 (1.8)	38 (1.0)	0.062	0.067	5824 (0.9)	12 (1.7)	5812 (0.9)	0.031	0.075
Cancer (excluded lung cancer)	264 (5.5)	66 (6.9)	198 (5.1)	0.044	0.072	35,026 (5.4)	63 (9.2)	34,963 (5.4)	<0.001	0.146
Cerebrovascular disease	201 (4.2)	67 (7.0)	134 (3.5)	<0.001	0.157	19,295 (3.0)	37 (5.4)	19,258 (3.0)	<0.001	0.121
Chronic obstructive pulmonary disease	180 (3.7)	63 (6.5)	117 (3.0)	<0.001	0.165	17,829 (2.7)	32 (4.7)	17,797 (2.7)	0.003	0.102
Congestive heart failure	420 (8.7)	151 (15.7)	269 (7.0)	<0.001	0.277	45,190 (7.0)	118 (17.2)	45,072 (7.0)	<0.001	0.318
Diabetes mellitus	1609 (33.5)	407 (42.3)	1202 (31.2)	<0.001	0.231	206,457 (31.8)	285 (41.5)	206,172 (31.8)	<0.001	0.203
Hypokalemia	31 (0.6)	15 (1.6)	16 (0.4)	<0.001	0.116	1987 (0.3)	13 (1.9)	1974 (0.3)	<0.001	0.153
Hypothyroidism	234 (4.9)	74 (7.7)	160 (4.2)	<0.001	0.150	27,365 (4.2)	46 (6.7)	27,319 (4.2)	0.002	0.110
Ischemic heart disease	709 (14.7)	206 (21.4)	503 (13.1)	<0.001	0.222	81,692 (12.6)	138 (20.1)	81,554 (12.6)	<0.001	0.205
Liver cirrhosis	28 (0.6)	9 (0.9)	19 (0.5)	0.169	0.052	1947 (0.3)	8 (1.2)	1939 (0.3)	<0.001	0.102
Lung cancer	32 (0.7)	11 (1.1)	21 (0.5)	0.069	0.065	3720 (0.6)	5 (0.7)	3715 (0.6)	0.775	0.019
Nocturia	101 (2.1)	57 (5.9)	44 (1.1)	<0.001	0.261	10,075 (1.6)	41 (6.0)	10,034 (1.5)	<0.001	0.234
Pancreatic disorder	34 (0.7)	17 (1.8)	17 (0.4)	<0.001	0.127	3933 (0.6)	12 (1.7)	3921 (0.6)	<0.001	0.106
Pneumonia	168 (3.5)	61 (6.3)	107 (2.8)	<0.001	0.171	16,959 (2.6)	44 (6.4)	16,915 (2.6)	<0.001	0.184
Renal failure	155 (3.2)	62 (6.4)	93 (2.4)	<0.001	0.197	17,105 (2.6)	40 (5.8)	17,065 (2.6)	<0.001	0.159
**Polypharmacy**				<0.001	0.682				<0.001	0.615
0–4	1394 (29.0)	129 (13.4)	1265 (32.9)			213,501 (32.9)	102 (14.9)	213,399 (32.9)		
5–9	1843 (38.3)	298 (31.0)	1545 (40.2)			256,989 (39.6)	224 (32.7)	256,765 (39.6)		
10–14	1054 (21.9)	304 (31.6)	750 (19.5)			125,466 (19.3)	192 (28.0)	125,274 (19.3)		
≥15	519 (10.8)	231 (24.0)	288 (7.5)			52,630 (8.1)	168 (24.5)	52,462 (8.1)		
**Number of HIMs**				<0.001	0.534				<0.001	0.530
0	2096 (43.6)	247 (25.7)	1849 (48.1)			317,235 (48.9)	196 (28.6)	317,039 (48.9)		
1	1339 (27.8)	272 (28.3)	1067 (27.7)			171,401 (26.4)	163 (23.8)	171,238 (26.4)		
2	596 (12.4)	174 (18.1)	422 (11.0)			72,969 (11.3)	123 (17.9)	72,846 (11.2)		
≥3	779 (16.2)	269 (28.0)	510 (13.3)			86,981 (13.4)	204 (29.7)	86,777 (13.4)		
**Patterns of HIM use**										
Anticonvulsants, new use	39 (0.8)	16 (1.7)	23 (0.6)	0.002	0.101	3769 (0.6)	5 (0.7)	3764 (0.6)	0.796	0.018
Anticonvulsants, persistent use	192 (4.0)	89 (9.3)	103 (2.7)	<0.001	0.280	18,361 (2.8)	49 (7.1)	18,312 (2.8)	<0.001	0.199
Antipsychotics, new use	19 (0.4)	5 (0.5)	14 (0.4)	0.687	0.024	1551 (0.2)	3 (0.4)	1548 (0.2)	0.501	0.034
Antipsychotics, persistent use	131 (2.7)	45 (4.7)	86 (2.2)	<0.001	0.134	16,898 (2.6)	48 (7.0)	16,850 (2.6)	<0.001	0.207
Desmopressin, new use	4 (0.1)	2 (0.2)	2 (0.1)	0.381	0.043	498 (0.1)	4 (0.6)	494 (0.1)	<0.001	0.089
Desmopressin, persistent use	61 (1.3)	41 (4.3)	20 (0.5)	<0.001	0.247	5150 (0.8)	31 (4.5)	5119 (0.8)	<0.001	0.234
Mirtazapine, new use	4 (0.1)	2 (0.2)	2 (0.1)	0.381	0.043	336 (0.1)	1 (0.1)	335 (0.1)	0.808	0.030
Mirtazapine, persistent use	33 (0.7)	17 (1.8)	16 (0.4)	<0.001	0.130	3852 (0.6)	8 (1.2)	3844 (0.6)	0.089	0.061
Opioids, new use	189 (3.9)	55 (5.7)	134 (3.5)	0.002	0.107	19,424 (3.0)	34 (5.0)	19,390 (3.0)	0.004	0.101
Opioids, persistent use	140 (2.9)	55 (5.7)	85 (2.2)	<0.001	0.181	14,212 (2.2)	43 (6.3)	14,169 (2.2)	<0.001	0.204
PPIs, new use	148 (3.1)	46 (4.8)	102 (2.7)	<0.001	0.113	20,451 (3.2)	28 (4.1)	20,423 (3.2)	0.199	0.050
PPIs, persistent use	585 (12.2)	184 (19.1)	401 (10.4)	<0.001	0.247	72,259 (11.1)	140 (20.4)	72,119 (11.1)	<0.001	0.257
SNRIs, new use	15 (0.3)	9 (0.9)	6 (0.2)	<0.001	0.106	1010 (0.2)	3 (0.4)	1007 (0.2)	0.165	0.052
SNRIs, persistent use	73 (1.5)	24 (2.5)	49 (1.3)	0.009	0.090	7061 (1.1)	13 (1.9)	7048 (1.1)	0.064	0.067
SSRIs, new use	23 (0.5)	11 (1.1)	12 (0.3)	0.002	0.098	2191 (0.3)	7 (1.0)	2184 (0.3)	0.006	0.083
SSRIs, persistent use	163 (3.4)	56 (5.8)	107 (2.8)	<0.001	0.150	20,077 (3.1)	44 (6.4)	20,033 (3.1)	<0.001	0.157
TCAs, new use	36 (0.7)	14 (1.5)	22 (0.6)	0.008	0.088	2899 (0.4)	7 (1.0)	2892 (0.4)	0.049	0.067
TCAs, persistent use	145 (3.0)	49 (5.1)	96 (2.5)	<0.001	0.136	15,677 (2.4)	25 (3.6)	15,652 (2.4)	0.049	0.072
Thiazide diuretics, new use	31 (0.6)	15 (1.6)	16 (0.4)	<0.001	0.116	3537 (0.5)	15 (2.2)	3522 (0.5)	<0.001	0.142
Thiazide diuretics, persistent use	829 (17.2)	246 (25.6)	583 (15.2)	<0.001	0.261	91,033 (14.0)	164 (23.9)	90,869 (14.0)	<0.001	0.254
**Concurrent use of HIMs**										
Desmopressin—Thiazide diuretics	10 (0.2)	9 (0.9)	1 (0.0)	<0.001	0.132	673 (0.1)	8 (1.2)	665 (0.1)	<0.001	0.134
Desmopressin—SIADH-inducing drug	29 (0.6)	18 (1.9)	11 (0.3)	<0.001	0.154	2042 (0.3)	20 (2.9)	2022 (0.3)	<0.001	0.208
Thiazide diuretics—SIADH-inducing drug	258 (5.4)	108 (11.2)	150 (3.9)	<0.001	0.280	23,430 (3.6)	81 (11.8)	23,349 (3.6)	<0.001	0.311
SIADH-inducing drug—SIADH-inducing drug	185 (3.8)	77 (8.0)	108 (2.8)	<0.001	0.231	19,893 (3.1)	66 (9.6)	19,827 (3.1)	<0.001	0.272

Abbreviations: CCI, Charlson Comorbidity Index; HIM, hyponatremia-inducing medication; HN, hyponatremia; PPIs, proton pump inhibitors; SIADH, syndrome of inappropriate antidiuretic hormone secretion; SMD, standardized mean difference; SNRIs, serotonin-norepinephrine reuptake inhibitors; SSRIs, selective serotonin reuptake inhibitors; TCAs, tricyclic antidepressants.

**Table 2:** Predictive performance of six machine learning models in development and validation cohorts.

Model	AUROC	(95% CI)	Brier Score	ACC	SEN	SPE	PPV	NPV	F1 Score
**Development cohort**									
Logistic Regression	0.7489	(0.717–0.780)	0.1990	0.7275	0.6585	0.7448	0.3921	0.8972	0.4915
LASSO	0.7461	(0.714–0.777)	0.1979	0.7345	0.6307	0.7605	0.3969	0.8917	0.4872
Random Forest	0.7542	(0.722–0.785)	0.1379	0.7373	0.6864	0.7500	0.4070	0.9054	0.5110
LightGBM	0.7488	(0.717–0.779)	0.1387	0.7199	0.6899	0.7273	0.3875	0.9037	0.4962
XGBoost	0.7565	(0.724–0.787)	0.1371	0.7038	0.7456	0.6934	0.3781	0.9160	0.5018
CatBoost	0.7525	(0.722–0.786)	0.1381	0.6885	0.7526	0.6725	0.3649	0.9158	0.4915
**Validation cohort**									
Logistic Regression	0.7443	(0.726–0.764)	0.1908	0.7379	0.6239	0.7380	0.0025	0.9995	0.0050
LASSO	0.7431	(0.725–0.760)	0.1891	0.7604	0.5860	0.7606	0.0026	0.9994	0.0051
Random Forest	0.7414	(0.723–0.759)	0.0397	0.7493	0.5918	0.7494	0.0025	0.9994	0.0050
LightGBM	0.7456	(0.728–0.763)	0.0417	0.7127	0.6341	0.7128	0.0023	0.9995	0.0046
XGBoost	0.7438	(0.723–0.762)	0.0426	0.6956	0.6472	0.6957	0.0022	0.9995	0.0045
CatBoost	0.7449	(0.728–0.762)	0.0419	0.6807	0.6691	0.6807	0.0022	0.9995	0.0044

Abbreviations. ACC, accuracy; AUROC, area under the receiver operating characteristic curve; CI, confidence interval; NPV, negative predictive value; PPV, positive predictive value; SEN, sensitivity; SPE, specificity.

## Data Availability

HIRA research data (M20200324406) analyzed in this study was provided by Health Insurance Review and Assessment Service in Republic of Korea. It is prohibited to transfer, rent or sell the research data to any third party other than the researcher who has been officially approved for database use. However, other researchers may request access to the data directly from the HIRA by submitting a research plan for review and obtaining approval from the HIRA data provision deliberation committee, in accordance with HIRA’s data access policy. The analytic code and trained model objects are available from the corresponding author on reasonable request and will be deposited in a public repository upon publication.

## Supplementary Materials

The following supporting information can be downloaded at: https://www.mdpi.com/article/10.3390/jcm15135072/s1, Table S1: Modified list of medications-inducing hyponatremia in Korea; Table S2: Explored hyperparameter fields and selected hyperparameters; Table S3, Subgroup performance of the LightGBM model on the 2019 temporal-validation cohort; Figure S1: SHAP-based interpretability for LightGBM and CatBoost models; Figure S2: Calibration of the LightGBM model on the full 2019 temporal-validation cohort before and after recalibration.

## Abbreviations

The following abbreviations are used in this manuscript:

ACC	accuracy
ADH	antidiuretic hormone
AGS	American Geriatrics Society
AUPRC	area under the precision–recall curve
AUROC	area under the receiver operating characteristic curve
BS	Brier score
CatBoost	categorical boosting
CCI	Charlson Comorbidity Index
CHF	congestive heart failure
CI	confidence interval
CITL	calibration-in-the-large
COPD	chronic obstructive pulmonary disease
DCA	decision curve analysis
EHR	electronic health record
EPV	events-per-variable
HIM	hyponatremia-inducing medication
HIRA-APS	Health Insurance Review and Assessment Service-Aged Patient Sample
HN	hyponatremia
ICD-10	International Classification of Diseases, 10th Revision
IHD	ischemic heart disease
IRB	Institutional Review Board
LASSO	least absolute shrinkage and selection operator
LightGBM	light gradient boosting machine
LR	logistic regression
ML	machine learning
NPV	negative predictive value
PIMs	potentially inappropriate medications
PPIs	proton pump inhibitors
PPV	positive predictive value
PR	precision–recall
RFECV	recursive feature elimination with cross-validation
ROC	receiver operating characteristic
SEN	sensitivity
SHAP	SHapley Additive exPlanations
SIADH	syndrome of inappropriate antidiuretic hormone secretion
SMD	standardized mean difference
SMOTE	synthetic minority over-sampling technique
SNRIs	serotonin-norepinephrine reuptake inhibitors
SPE	specificity
SSRIs	selective serotonin reuptake inhibitors
TCAs	tricyclic antidepressants
XGBoost	extreme gradient boosting
