# Supplementary material for: Development and Temporal Validation of Machine Learning Models for Hyponatremia Risk in Community-Dwelling Older Adults: A Nationwide Claims-Based Study
Source: J Clin Med. 2026 Jun 29;15(13):5072. doi: 10.3390/jcm15135072 (PMC13363158; doi:10.3390/jcm15135072)
Supplement: Supplementary file 1 [file jcm-15-05072-s001.zip › Supplementary Materials Tables.pdf]

## Supplementary table legends

Table S1. Modified list of medications-inducing hyponatremia in Korea

Table S2. Explored hyperparameter fields and selected hyperparameters

Table S3. Subgroup performance of the LightGBM model on the 2019 temporal-validation cohort

**Table S1.** Modified list of medications-inducing hyponatremia in Korea

| Class | Medication (ATC codes*) |
|---|---|
| **Exogenous antidiuretic hormone analogs** | |
| Desmopressin (H01BA02) | |
| **Medication that alters sodium and water homeostasis** | |
| Thiazide diuretics | chlorthalidone (C03BA04), hydrochlorothiazide (C03AA03), indapamide (C03BA11), metolazone (C03BA08), xipamide (C03BA10) |
| **Medications causing SIADH** | |
| Anticancer | carboplatin (L01XA02), cisplatin (L01XA01), cyclophosphamide (L01AA01), vincristine (L01CA02) |
| Anticonvulsants | carbamazepine (N03AF01), gabapentin (N03AX12), lamotrigine (N03AX09), levetiracetam (N03AX14), oxcarbazepine (N03AF02), phenytoin (N03AB02), valproic acid /sodium valproate (N03AG01), topiramate (N03AX11) |
| Antipsychotics | amisulpride (N05AL05), aripiprazole (N05AX12), blonanserin (N05AX), chlorpromazine (N05AA01), chlorprothixene (N05AF03), clozapine (N05AH02), haloperidol (N05AD01), levomepromazine (N05AA02), lithium (N05AN01), olanzapine (N05AH03), paliperidone (N05AX13), perphenazine (N05AB03), pimozide (N05AG02), quetiapine (N05AH04), risperidone (N05AX08), sulpiride (N05AL01), ziprasidone (N05AE04), zotepine (N05AX11) |
| Opioids | alfentanil (N01AH02), buprenorphine (N02AE01), butorphanol (N02AF01), codeine (R05DA04, N02AJ09), dihydrocodeine (N02AA08, N02AA58), fentanyl (N02AB03), hydromorphone (N02AA03), morphine (N02AA01), nalbuphine (N02AF02), oxycodone (N02AA05, N02AA55), pethidine (N02AB02), remifentanil (N01AH06), sufentanil (N01AH03), tapentadol (N02AX06), tramadol (N02AX02) |
| PPIs | dexlansoprazole (A02BC06), esomeprazole (A02BC05), ilaprazole (A02BX), lansoprazole (A02BC03), omeprazole (A02BC01), pantoprazole (A02BC02), rabeprazole (A02BC04), revaprazan (A02BC) |
| SNRIs | desvenlafaxine (N06AX23), duloxetine (N06AX21), milnacipran (N06AX17), venlafaxine (N06AX16) |
| SSRIs | citalopram (N06AB04), escitalopram (N06AB10), fluoxetine (N06AB03), fluvoxamine (N06AB08), paroxetine (N06AB05), sertraline (N06AB06) |
| TCAs | amitriptyline (N06AA09), amoxapine (N06AA17), clomipramine (N06AA04), dothiepin (N06AA16), doxepin (N06AA12), imipramine (N06AA02), nortriptyline (N06AA10) |
| Others | mirtazapine (N06AX11) |

*Abbreviations: PPIs, proton pump inhibitors; SNRIs, serotonin-norepinephrine reuptake inhibitors; SSRIs, selective serotonin reuptake inhibitors; TCAs, tricyclic antidepressants;*

**The WHO Anatomical Therapeutic Chemical Classification System (ATC)*

**Table S2.** Explored hyperparameter fields and selected hyperparameters

| Model | Search Range | Selected hyperparameters |
|---|---|---|
| Logistic regression | 'C': [0.000001, 1000000] | C=0.000494 |
| LASSO | 'C': [0.000001, 1000000] | C=0.244200 |
| Random Forest | 'n_estimators': [100, 600], 'max_depth': [5, 25], 'min_samples_split': [2, 10], 'min_samples_leaf': [1, 10], 'max_features': ['sqrt', 'log2'], 'bootstrap': [True, False] | n_estimators=202, max_depth=5, min_samples_split=4, min_samples_leaf=4, max_features='sqrt', bootstrap=True |
| LightGBM | 'n_estimators': [100, 600], 'learning_rate': [0.01, 0.3], 'max_depth': [3, 20], 'num_leaves': [20, 3000], 'min_child_samples': [5, 100], 'subsample': [0.5, 1.0], 'colsample_bytree': [0.5, 1.0], 'reg_alpha': [0.0, 10.0], 'reg_lambda': [0.0, 10.0] | n_estimators=370, learning_rate=0.2443, max_depth=16, num_leaves=2231, min_child_samples=18, subsample=0.5671, colsample_bytree=0.6667, reg_alpha=6.5295, reg_lambda=7.8143 |
| XGBoost | 'n_estimators': [100, 600], 'learning_rate': [0.01, 0.3], 'max_depth': [3, 20], 'subsample': [0.5, 1.0], 'colsample_bytree': [0.5, 1.0], 'gamma': [0.0, 10.0], 'reg_alpha': [0.0, 10.0], 'reg_lambda': [0.0, 10.0] | n_estimators=298, learning_rate=0.2267, max_depth=5, subsample=0.7242, colsample_bytree=0.7512, gamma=5.2267, reg_alpha=0.2051, reg_lambda=2.7458 |
| CatBoost | 'depth': [4, 10], 'learning_rate': [0.01, 0.3], 'iterations': [300, 1000], 'l2_leaf_reg': [2, 10], 'colsample_bylevel': [0.6, 1.0], 'subsample': [0.6, 1.0] | depth=4, learning_rate=0.0143, iterations=354, l2_leaf_reg=8, colsample_bylevel=0.7383, subsample=0.6763 |

**Table S3.** Subgroup performance of the LightGBM model on the 2019 temporal-validation cohort

| Subgroup | Patients, n | Events, n | AUROC (95% CI) | CITL |
|---|---|---|---|---|
| **Overall** | 648,586 | 686 | 0.746 (0.728–0.763) | ≈0 |
| **Sex** | | | | |
| Male | 271,727 | 239 | 0.737 (0.703–0.768) | −0.15 |
| Female | 376,859 | 447 | 0.748 (0.726–0.768) | 0.09 |
| **Age, years** | | | | |
| 65–69 | 205,307 | 69 | 0.751 (0.685–0.808) | −0.43 |
| 70–74 | 165,082 | 129 | 0.670 (0.618–0.717) | 0.10 |
| ≥75 | 278,197 | 488 | 0.675 (0.651–0.699) | 0.05 |
| **Insurance type** | | | | |
| Health insurance | 593,445 | 589 | 0.747 (0.727–0.766) | 0.00 |
| Medical aid | 46,001 | 82 | 0.669 (0.612–0.722) | 0.02 |
| National meritorious service [a] | 9,140 | 15 | 0.840 (0.718–0.915) | −0.16 |
| **CCI score** | | | | |
| 0 – 2 | 503,497 | 405 | 0.734 (0.710–0.757) | −0.02 |
| 3 – 4 | 111,536 | 187 | 0.707 (0.671–0.741) | −0.06 |
| ≥ 5 | 33,553 | 94 | 0.665 (0.607–0.719) | 0.25 |
| **Frailty score** | | | | |
| 0 ≤ score ≤ 2 | 432,972 | 308 | 0.718 (0.690–0.744) | −0.06 |
| 2 < score ≤ 5 | 158,350 | 219 | 0.713 (0.679–0.745) | −0.04 |
| > 5 | 57,264 | 159 | 0.691 (0.650–0.729) | 0.19 |
| **Medication burden (7-day)** | | | | |
| 0 – 4 | 265,922 | 128 | 0.704 (0.661–0.743) | 0.09 |
| 5 – 9 | 220,183 | 202 | 0.671 (0.634–0.705) | 0.02 |
| ≥ 10 | 162,481 | 356 | 0.696 (0.670–0.720) | −0.05 |

Abbreviations. AUROC, area under the receiver operating characteristic curve; CCI, Charlson Comorbidity Index; CI, confidence interval; CITL, calibration-in-the-large; SES, socioeconomic status.
95% CIs for AUROC were computed by the DeLong method. CITL is on the log-odds scale after a single global intercept recalibration; values near 0 indicate good calibration.
[a] Based on <30 events; the estimate is imprecise and shown for completeness only.
