# Supplementary figures and images for: Development and Temporal Validation of Machine Learning Models for Hyponatremia Risk in Community-Dwelling Older Adults: A Nationwide Claims-Based Study
Source: J Clin Med. 2026 Jun 29;15(13):5072. doi: 10.3390/jcm15135072 (PMC13363158; doi:10.3390/jcm15135072)

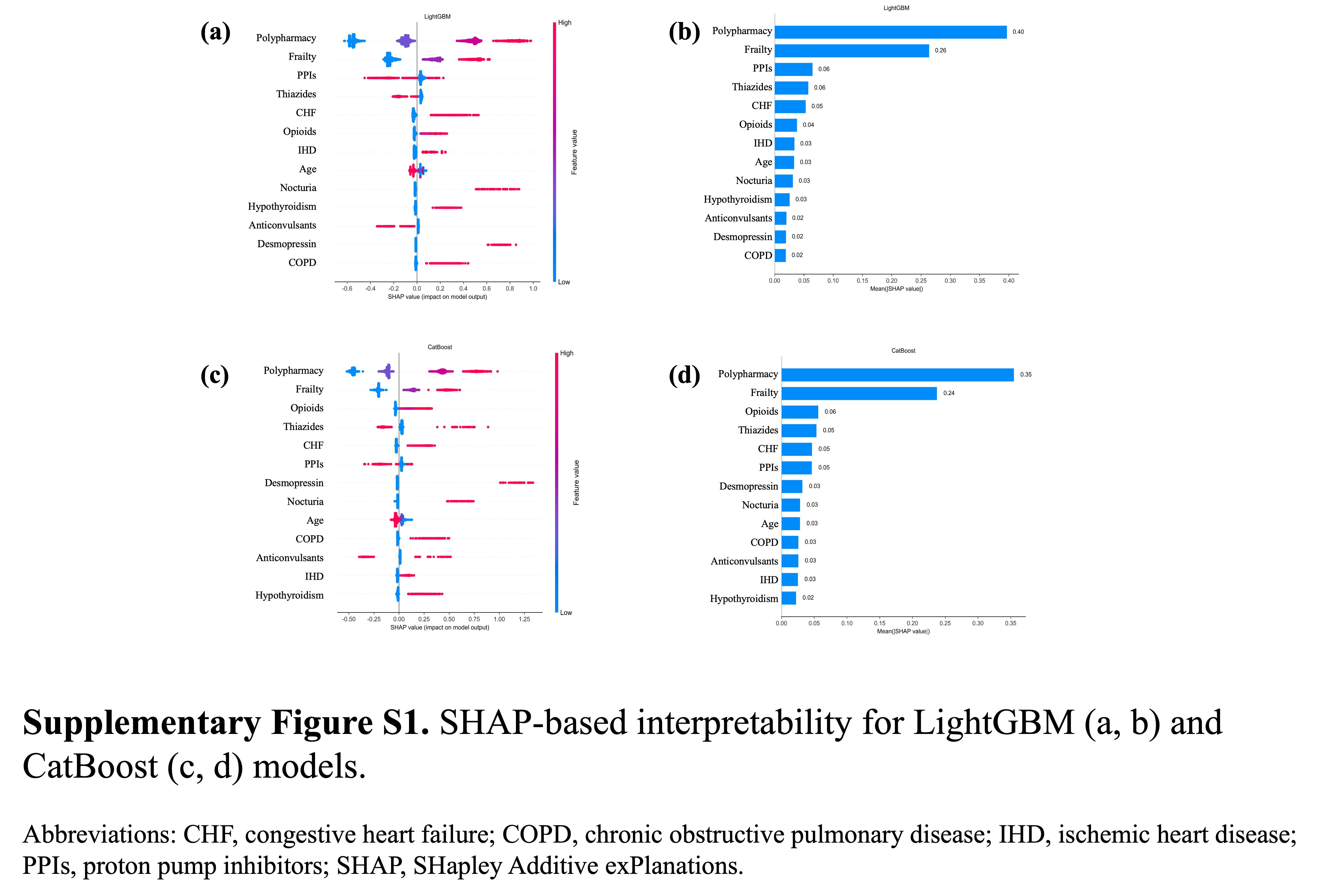

Supplement: Supplementary file 1 [file jcm-15-05072-s001.zip › Figure S1.jpg]

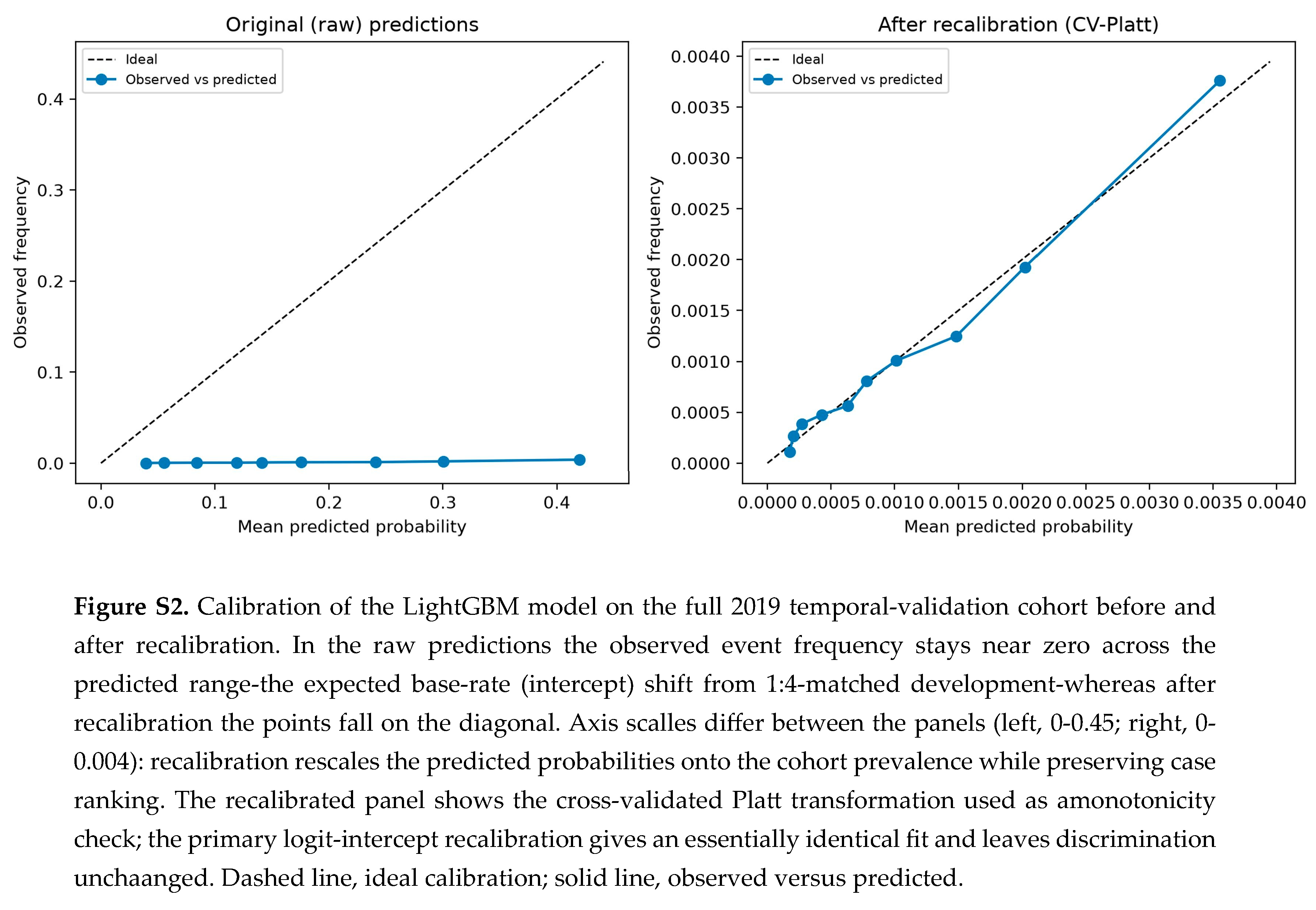

Supplement: Supplementary file 1 [file jcm-15-05072-s001.zip › Figure S2.png]
